# Definition of the viral targets of protective HIV-1-specific T cell responses

**DOI:** 10.1186/1479-5876-9-208

**Published:** 2011-12-07

**Authors:** Beatriz Mothe, Anuska Llano, Javier Ibarrondo, Marcus Daniels, Cristina Miranda, Jennifer Zamarreño, Vanessa Bach, Rosario Zuniga, Susana Pérez-Álvarez, Christoph T Berger, Maria C Puertas, Javier Martinez-Picado, Morgane Rolland, Marilu Farfan, James J Szinger, William H Hildebrand, Otto O Yang, Victor Sanchez-Merino, Chanson J Brumme, Zabrina L Brumme, David Heckerman, Todd M Allen, James I Mullins, Guadalupe Gómez, Philip J Goulder, Bruce D Walker, Jose M Gatell, Bonaventura Clotet, Bette T Korber, Jorge Sanchez, Christian Brander

**Affiliations:** 1 Irsicaixa AIDS Research Institute-HIVACAT, Badalona, Spain; 2 'Lluita contra la SIDA' Foundation, Hospital Germans Trias i Pujol, Badalona, Spain; 3 Universitat Autònoma de Barcelona, Barcelona, Spain; 4 Los Alamos National Laboratory, Los Alamos, NM, USA; 5 Asociación Civil IMPACTA Salud y Educacion, Lima, Peru; 6 Dept. Estadística i Investigació Operativa, Universitat Politècnica de Catalunya, Barcelona, Spain; 7 Ragon Institute of MGH, Harvard and MIT, Boston, MA, USA; 8 Institucio Catalana de Recerca i Estudis Avançats (ICREA), Barcelona, Spain; 9 MHRP, Frederick, USA; 10 University of Oklahoma Medical Center, Oklahoma City, OK, USA; 11 University of California, Los Angeles, CA, USA; 12 Services of Immunology and Institut d'Investigacions Biomediques August Pi i Sunyer (IDIBAPS)-AIDS Research Group-HIVACAT, Hospital Clinic, Barcelona, Spain; 13 British Columbia Centre for Excellence in HIV/AIDS, Vancouver, BC, Canada; 14 Simon Fraser University, Burnaby, BC, Canada; 15 Miscrosoft Research, Redmond, WA, USA; 16 Department of Microbiology, University of Washington, Seattle, WA, USA; 17 Department of Paediatrics, Nuffield Department of Medicine, Oxford, UK; 18 University of KwaZulu-Natal, HIV Pathogenesis Program, DDMRI, Durban, South Africa; 19 Howard Hughes Medical Institute, Chevy Chase, MD, USA; 20 Santa Fe Institute, Santa Fe, NM, USA

**Keywords:** HIV specific CTL, clade B, clade C, HLA, vaccine immunogen design, functional avidity, epitope, entropy, immune correlate

## Abstract

**Background:**

The efficacy of the CTL component of a future HIV-1 vaccine will depend on the induction of responses with the most potent antiviral activity and broad HLA class I restriction. However, current HIV vaccine designs are largely based on viral sequence alignments only, not incorporating experimental data on T cell function and specificity.

**Methods:**

Here, 950 untreated HIV-1 clade B or -C infected individuals were tested for responses to sets of 410 overlapping peptides (OLP) spanning the entire HIV-1 proteome. For each OLP, a "protective ratio" (PR) was calculated as the ratio of median viral loads (VL) between OLP non-responders and responders.

**Results:**

For both clades, there was a negative relationship between the PR and the entropy of the OLP sequence. There was also a significant additive effect of multiple responses to beneficial OLP. Responses to beneficial OLP were of significantly higher functional avidity than responses to non-beneficial OLP. They also had superior in-vitro antiviral activities and, importantly, were at least as predictive of individuals' viral loads than their HLA class I genotypes.

**Conclusions:**

The data thus identify immunogen sequence candidates for HIV and provide an approach for T cell immunogen design applicable to other viral infections.

## Background

HIV-1 infection induces strong and broadly directed HLA class I restricted T cell responses for which specific epitopes and restricting HLA class I alleles have been associated with relative in vivo viral control [1]. The bulk of the anti-viral CTL response appears to be disproportionately HLA-B restricted, but the relative contribution of targeted viral regions and restricting HLA molecules on the effectiveness of these responses remains unclear [2-5]. In addition, the impact of HIV-1 sequence diversity on the effectiveness of virus-specific T cell immunity in vivo is unclear, as functional constraints of escape variants, codon-usage at individual protein positions, T cell receptor (TCR) plasticity and functional avidity and cross-reactivity potential may all contribute to the overall antiviral activity of a specific T cell response [6-13]. Of note, T cell responses to Gag have most consistently been associated with reduced viral loads in both clade B and clade C infected cohorts [14-16]; however, the specific regions in Gag responsible for this effective control remain poorly defined. In addition, it is unclear whether the relative benefit of Gag is due to any other specific characteristic of this protein, such as rapid antigen-representation upon infection, protein expression levels, amino acid composition and/or inherently greater processability and immunogenicity, particularly in the context of selected HLA class I alleles [17,18]. Thus, concerns remain that a purely Gag-based vaccine might mainly benefit those people with a particular HLA genotype and will not take advantage of potentially beneficial targets outside of Gag [4,16,17,19]. In addition, CTL escape and viral fitness studies have focused largely on Gag-derived epitopes presented in the context of protective HLA class I alleles such as HLA-B27 and -B57 [7,20,21], yielding results that may not be generalizable to the genetically diverse majority of the human population. Furthermore, many studies have focused on immunodominant targets only, despite some studies in HIV-1 and SIV infection demonstrating a crucial contribution of sub-dominant responses to targets outside of Gag to the effective in-vivo viral control [4,22]. Thus, the current view on what may constitute a protective cellular immune response to HIV-1 is likely biased towards a immunodominant responses and those restricted by frequent HLA class I alleles and HLA alleles associated with superior disease outcome.

To overcome these potential limitations, the design of an effective and broadly applicable HIV-1 vaccine should to be based on information gained through comprehensive analyses that extend across large portions of the population's HLA class I heterogeneity. Here we focus on three cohorts totaling more than 950 untreated, chronically HIV-1 infected individuals with clade B and C infections, from which responses to certain regions of the viral genome and specific T cell response patterns emerge as correlates of viral control. Importantly, the analyses identify functional properties unique to these responses and control for the impact of HLA class I alleles known to be associated with superior control of HIV-1 infection, thus providing vaccine immunogen sequence candidates with potential usefulness in a broadly applicable HIV-1 vaccine.

## Methods

### Cohorts

A HIV clade B infected cohort of 223 chronically infected, treatment naïve individuals was recruited and tested at IMPACTA in Lima, Peru. The majority (78%) of enrollees were male and all recruited individuals considered themselves to be of a mixed Amerindian ethnicity [14]. The cohort had a median viral load 37,237 copies/ml (range < 50- > 750,000) and a median CD4 count of 385 cell/ul (range170-1151). A second clade B infected cohort was established at the HIV-1 outpatient clinic "Lluita contra la SIDA" at Hospital Germans Trias i Pujol in Badalona (Barcelona, Spain) consisting of 48 treatment-naïve subjects with viral loads below 10,000 and CD4 cell counts > 350 cells/mm^3 ^("controllers", n = 24) or above 50,000 copies/ml and CD4 cell counts < 350 cells/mm^3 ^("non-controllers", n = 24). The HIV-1 clade C infected cohort has been described in the past and consisted of 631 treatment naïve South African with a median viral load of 37,900 copies/ml (range < 50-> 750,000) and a median CD4 count of 393 cells/ul (range 1-1378) [16]. An additional 78 from a recently published cohort in Boston were included in the analyses of functional avidities [23-29]. HLA typing was performed as previously described using SSP-PCR [30]. For Hepitope and FASS analyses, 4digit typing was used for the Lima cohort and 2-digit typing for the Durban cohort. Protocols were approved in Lima by the IMPACTA Human Research Committee, in Durban by the Ethical Committee of the Nelson R. Mandela School of Medicine at the University of KwaZulu-Natal and in Barcelona by the Human Research Committee at Hospital Germans Trias i Pujol. All subjects provided written informed consent.

Peptide test set and ELISpot assay: Previously described peptide sets matching HLA-clade B and C consensus sequences were used in all experiments for which the OLP-specific entropies have been calculated in the past, based on available sequence datasets [31-33] and http://www.hiv.lanl.gov/content/immunology/hlatem/index.html. The peptides were clade-specific sets of adapted 18mers, overlapping by 11 residues designed using the PeptGen tool available at the Los Alamos HIV database http://www.hiv.lanl.gov/content/sequence/PEPTGEN/peptgen.html. The individual OLP in the peptide sets for clade B and clade C had all the same starting and ending position relative to the source protein and follow the same numbering across the entire viral proteome for both clades. Peripheral blood mononuclear cells (PBMCs) were separated from whole blood by density centrifugation and used directly to test for CD8^+ ^T cell responses in vitro. IFN-γ ELISpot assays were performed as described previously, using Mabtech antibodies (Mabtech, Stockholm, Sweden) and a matrix format that allowed simultaneous testing of all 410 overlapping (OLP) peptides in the respective test set [14]. Thresholds for positive responses were defined as: exceeding 5 spots (50 SFC/10^6^) per well and exceeding the mean of negative wells plus 3 standard deviation or three times the mean of negative wells, whichever was higher. Stimulation with PHA was used as a positive control in all ELISpot assays.

### Definition of functional avidity

Responses targeting 18 mer OLP in HIV-1 Gag p24 were assessed for their functional avidity using OLP-specific sets of 10 mer peptides overlapping by 9 residues that span the 18 mer peptide sequence. Functional avidity was defined as the peptide concentration needed to elicit half maximal response rates in the ELISpot assay and was calculated as a sigmoidal dose response curve fit using GraphPad Prism software [13].

### *In vitro *viral replication inhibition assay

A double mutant virus containing a Nef M20A and Integrase G140S/Q148H Raltegravir (integrase inhibitor) resistance mutations was tested for replication in CD4 T cells in the presence or absence of autologous T cell lines targeting protective or non-protective OLP. Use of the Raltegravir-resistant virus allows to prevent potential replication of autologous virus in the inhibition assays [28], excludes potential negative impacts on antigen processing or CTL functions attributed to protease inhibitors [34] and avoids overlap between the resistance mutations sites (i.e. G140S/Q148H) and location of beneficial and non-beneficial OLP sequences. In brief, the p83-10 plasmid containing mutations for a methionine to alanine substitution at position 20 of the Nef protein and the p83-2 plasmid engineered to contain the G140S and Q148H mutations in the integrase were combined to produce a virus that is replication competent, highly resistant to Raltegravir and does not downregulate HLA class I in infected cells [35,36]. Although not entirely physiological, this approach was chosen to potentially increase the signal in the in vitro inhibition assay, even when responses were restricted by Nef-sensitive HLA class I alleles. Plasmids were co-transfected into MT4 cells and virus was harvested after 7 days [35,37,38]. Autologous CD4 cells were enriched by magnetic beads isolation (Miltenyi) and expanded for 3 days using a bi-specific anti-CD3/8 antibody and IL-2 containing medium (50 IU r-IL2) before infecting them at multiplicities of infection (MOI) between 0.01 and 1. Effector cells were obtained by stimulating PBMC with either beneficial or non-beneficial OLP for 12 days before isolating specific OLP-reactive cells by IFN-γ capture assay according to manufacturers' instructions (Miltenyi, Bergisch Gladbach, Germany). The effector T cells were analyzed by flow cytometry for the specificity to their respective targets after capture assay and quantified to adjust effector-to-target ratios. Since the NL4-3 backbone sequence differed in several positions in beneficial and non-beneficial OLP, the epitope specificity was predicted based on the HLA class I genotype of the tested individual and responses confirmed to efficiently recognize variant sequences in the NL4-3 backbone sequence. Culture supernatant was harvested and replaced by Raltegravir containing medium 0.05 μg/ml after 72 h. Levels of Gagp24 in the culture supernatant were determined by ELISA as described [39].

### Statistical Analyses

Statistical analyses were performed using Prism Version 5 and R Statistical Language [40]. Results are presented as median values unless otherwise stated. Tests included ANOVA, non-parametric Mann-Whitney test (two-tailed) and Spearman rank test. The significance of differences in viral load distribution between OLP-responders and OLP-non-responders was assessed by a two-sided Student's T Test with multiple tests addressed using, instead of a Bonferroni correction, a q-value approach to compensate for multiple comparisons [39]. The multivariate analysis was based on a novel multivariate combined regression method known as FASS, a forward selection method combined with all-subsets regression [41-43]. Briefly, the FASS approach works by iteratively performing the following procedure: Let 'V' be the set of all variables and 'M' be the set of variables included in a model. In the first step, those variables that are not already in the model are divided into equal-sized blocks of variables (the last block may have less than 'g' variables). Then, for each block of variables, 'm' is a new estimated and evaluated model using the Bayesian Information Criterion (BIC). The best model 'm' according to its BIC is retained and the procedure starts all over again until in one step or more the model is not improved.

## Results

### HIV-1-specific T cell responses targeting conserved regions are associated with lower viral loads

**Figure 1:**
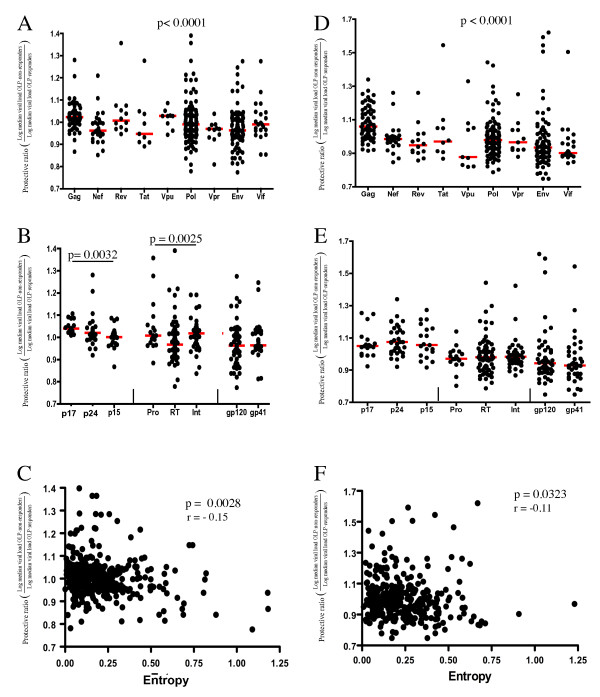
**Localization and conservation of beneficial and non-beneficial OLP in HIV-1 clade B and C cohorts**. Total HIV-1-specific T cell responses were assessed in a cohort of 223 chronically HIV clade B infected, untreated individuals in Lima, Peru (**graphs A-C**) and in 631 chronically HIV clade C infected, untreated individuals in Durban, South Africa (**graphs D-F**) using peptide test sets of 410 18 mer overlapping peptides (OLP) spanning the consensus B and C sequences, respectively [2,31]. For each OLP, the protective ratio (PR, defined as "the ratio of the log median viral load in OLP non-responders divided by log median viral load in OLP responders") was determined. Each symbol represents an individual OLP, grouped either by (**A, D**) proteins or (**B, E**) protein-subunits for OLP located in Gag, Pol and Env (p-values in A, D based on ANOVA, in B, E on Mann-Whitney by pariwise comparing the different protein subunits, red lines indicating median PR values). In (**C and F**), the OLP-specific entropy (a measure of the viral diversity in the region the OLP spans) is compared to the OLP-specific PR and shows an inverse association between the sequence conservation and PR (Spearman rank).

In a first analysis, HIV-1-specific T cell responses were assessed in a cohort of 223 HIV-1 clade B infected individuals recruited in Lima, Peru using IFNg ELISpot assays and a previously described set of 410 clade B overlapping peptides (OLP) [14,31]. For each OLP, a protective ratio (PR) was calculated as the ratio of the median viral loads between OLP non-responders and OLP responders, such that OLP with PR > 1 were reflective of OLP predominantly targeted by individuals with reduced viral loads. OLP-specific PR were a) compared between OLP spanning the different viral proteins and b) correlated with the viral sequence heterogeneity in the region covered by the OLP. The data showed highest median PR values for OLP spanning the Gag protein sequence, whereas Nef, Env and Tat had the lowest median PR values (Figure 1A, p < 0.0001, ANOVA). A protein-subunit-breakdown of PR values showed the p15 subunit of Gag and RT in Pol to score less favorable than the remainder of the respective proteins (Figure 1B, p = 0.0032 and p = 0.0025, respectively). While these data confirm the association between HIV-1 Gag-specific responses and lower viral loads, it is important to note that all proteins contained OLP with PR > 1, suggesting that some beneficial responses can be located outside of Gag; data that has not emerged from any of the previous studies linking Gag responses to relative viral control. At the same time, all proteins contained OLP with PR < 1, indicating that proteins considered overall beneficial may contain non-beneficial regions as well. In addition, when the OLP-specific PR was compared to the sequence entropy of the region spanned by the individual OLP, a significant negative correlation between PR and entropy was observed (p = 0.0028, r = -0.15; Figure 1C). Although rarely targeted OLP may have introduced statistically less robust data points in this comparison and caused a wide scatter of data points, the results show a relative absence of OLP with high entropy and high PR values, suggesting that responses to more variable regions are less effective in mediating in vivo viral control.

To assess whether the above observations would also hold true outside of clade B infection, the same analyses were conducted in a cohort of 631 clade C HIV-1 infected subjects enrolled in Durban, South Africa and tested for responses against a clade C consensus OLP sequence as described previously [33]. As in clade B infection, the OLP specific PR values were highest for OLP spanning Gag without any significant differences between the Gag and Pol protein subunits (Figure 1D and 1E). As in the clade B cohort, the PR values were negatively correlated with the OLP-specific entropy (p = 0.0323, Figure 1F), confirming the findings in the clade B cohort and further pointing towards the importance of targeting conserved segments of the viral proteome for effective in vivo viral control.

### Identification of individual beneficial OLP sequences in clade B and C infection

**Figure 2:**
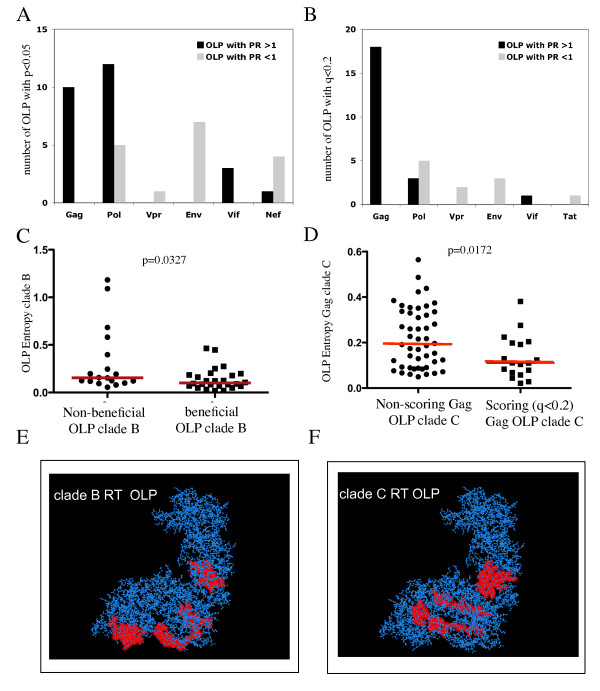
**Genome distribution, entropy and RT localization of OLP with significant impact on viral loads in HIV-1 clade B and C infection**: The distribution of OLP with significantly elevated or reduced PR across the viral proteome is shown in **A) **for clade B infection (cut-off uncorrected p-value of p < 0.05) and in **B) **for clade C infection (cut-off q < 0.2). The entropy of beneficial and non-beneficial clade B OLP is compared in **C) **while in **D)**, the entropy of beneficial OLP in HIV clade C Gag is compared to the remainder of Gag OLP (p-values based on Mann Whitney, red lines indicating median sequence entropies). In E and F, protein structures for HIV-1 reverse transcriptase (Protein databank structure ID 3IG1) were loaded into the Los Alamos HIV Database "protein feature accent" tool http://www.hiv.lanl.gov/content/sequence/PROTVIS/html/protvis.html and locations of beneficial RT OLP identified in clade B (Table 1) and in clade C (Table 2) marked by red highlights.

In order to identify individual OLP that were significantly more frequently targeted in individuals with relative viral control and to compare the beneficial OLP in clade B and C infection, the viral load distribution in OLP-responders and non-responders was analyzed individually for each OLP. For the clade B cohort in Peru, the analyses yielded 43 OLP sequences for which the median viral load differed between the two groups with an uncorrected p-value of < 0.05. Of these 43 OLP, 26 were OLP with a PR > 1 (referred to as "beneficial" OLP), and 17 OLP with a PR < 1 ("non-beneficial" OLP, Table 1). The distribution of OLP with PR > 1 among viral proteins was biased towards Gag and Pol, while Env produced exclusively OLP with PR < 1 (Figure 2A).

**Table 1:** Beneficial and non-beneficial OLP identified in Lima clade B cohort (p < 0.05)

OLP #	Protein	Sub-unit	OLP clade B consensus sequence	Median viral load in OLP responders	Median viral load in OLP non-responders	Protective Ratio (PR)*	p-value
3	Gag	p17	EKIRLRPGGKKKYKLKHI	22947	39014	**1.053**	0.037
6	Gag	p17	ASRELERFAVNPGLL	15380	43189	**1.107**	0.001
7	Gag	p17	ERFAVNPGLLETSEGCR	25939	38974	**1.040**	0.049
10	Gag	p17	QLQPSLQTGSEELRSLY	16285	37237	**1.085**	0.031
12	Gag	p17	SLYNTVATLYCVHQRIEV	23855	37113	**1.044**	0.037
23	Gag	p24	AFSPEVIPMFSALSEGA	22947	37113	**1.048**	0.036
31	Gag	p24	IAPGQMREPRGSDIA	3563	35483	**1.281**	0.028
34	Gag	p24	STLQEQIGWMTNNPPIPV	6127	37360	**1.207**	0.002
48	Gag	p24	ACQGVGGPGHKARVLAEA	12975	35755	**1.107**	0.041
60	Gag	p15	GKIWPSHKGRPGNFLQSR	16266	36434	**1.083**	0.044
75	Nef	-	WLEAQEEEEVGFPVRPQV	13407	37360	**1.108**	0.026
76	Nef	-	EVGFPVRPQVPLRPMTYK	59618	29855	0.937	0.001
84	Nef	-	NYTPGPGIRYPLTFGWCF	55402	30538	0.945	0.006
85	Nef	-	RYPLTFGWCFKLVPV	69890	29903	0.924	0.002
90	Nef	-	SLHGMDDPEKEVLVWKF	89687	32650	0.911	0.042
159	Pol	Pro	KMIGGIGGFIKVRQYDQI	14736	36434	**1.094**	0.020
160	Pol	Pro	FIKVRQYDQILIEICGHK	3682	35755	**1.277**	0.031
161	Pol	Pro	QILIEICGHKAIGTVLV	9117	35483	**1.149**	0.050
163	Pol	Pro	LVGPTPVNIIGRNLLTQI	25965	45637	**1.055**	0.007
171	Pol	RT	LVEICTEMEKEGKISKI	1865	35483	**1.391**	0.014
181	Pol	RT	LDVGDAYFSVPLDKDFRK	65858	32871	0.937	0.041
195	Pol	RT	LRWGFTTPDKKHQKEPPF	5624	37113	**1.219**	0.006
196	Pol	RT	DKKHQKEPPFLWMGYELH	10103	35483	**1.136**	0.044
210	Pol	RT	EIQKQGQGQWTYQIY	18155	35483	**1.068**	0.045
222	Pol	RT	PPLVKLWYQLEKEPIVGA	412599	34640	0.808	0.030
230	Pol	RT	IHLALQDSGLEVNIV	85102	34117	0.919	0.030
237	Pol	RT	VYLAWVPAHKGIGGNEQV	85102	34117	0.919	0.029
240	Pol	RT	SAGIRKVLFLDGIDKA	116902	32761	0.891	0.019
269	Pol	Int	TKELQKQITKIQNFRVYY	6629	35755	**1.192**	0.030
270	Pol	Int	TKIQNFRVYYRDSRDPLW	18171	37360	**1.073**	0.019
271	Pol	Int	YYRDSRDPLWKGPAKLLW	25939	35755	**1.032**	0.043
276	Pol	Int	KIIRDYGKQMAGDDCVA	6629	35755	**1.192**	0.021
279	Vpr	-	GPQREPYNEWTLELLEEL	60222	32650	0.944	0.042
307	Env	Gp120	DLNNNTNTTSSSGEKMEK	179419	34117	0.863	0.044
311	Env	Gp120	IRDKVQKEYALFYKLDVV	179419	32871	0.860	0.008
314	Env	Gp120	YRLISCNTSVITQACPKV	58206	31273	0.943	0.008
315	Env	Gp120	SVITQACPKVSFEPIPIH	61011	32871	0.944	0.034
320	Env	Gp120	TNVSTVQCTHGIRPVV	341587	34640	0.820	0.034
355	Env	Gp120	VAPTKAKRRVVQREKRAV	161602	34117	0.870	0.042
399	Env	Gp41	VIEVVQRACRAILHIPRR	388089	34640	0.812	0.026
405	Vif	-	VKHHMYISGKAKGWFYRH	16458	37237	**1.084**	0.021
406	Vif	-	GKAKGWFYRHHYESTHPR	16458	37237	**1.084**	0.022
424	Vif	-	TKLTEDRWNKPQKTKGHR	10319	36434	**1.137**	0.014

* PR values in bold indicate PR > 1, i.e. OLP-responses seen more frequently in individuals with reduced viral loads

The same analyses were repeated for the clade C cohort in Durban, which due to its larger size allowed to apply more stringent statistical criteria to identify beneficial and non-beneficial OLP. To compensate for multiple statistical comparisons, we employed a previously described false-discovery rate approach [39], resulting in the identification of 33 clade C OLP with q-values of < 0.2 (i.e. OLP with significantly different viral load distributions between OLP-responders and non-responders with a false positive discovery rate (q-value) of 20%). The 33 OLP identified were comprised of 22 beneficial OLP and 11 non-beneficial OLP, with the beneficial OLP being again located in Gag, Pol and Vif, similar to what was seen in the clade B cohort (Figure 2B).

In both cohorts, the total breadth and magnitude of responses did not correlate with viral loads as reported for parts of these cohorts in the past [14,16]. The OLP with significant differences in median viral loads (43 OLP in clade B and 33 OLP in clade C, Tables 1 and 2, respectively, i.e. "scoring OLP"), were more often targeted in their respective cohort than OLP that did not score with a significant difference in viral loads (p = 0.0015 Lima; p < 0.0001 Durban). However, beneficial and non-beneficial OLP were equally frequently targeted in either cohort. Also, there was no difference in the median magnitude of the OLP-specific responses, regardless whether it was a beneficial, non-beneficial or not-scoring OLP (all p > 0.7, data not shown). Finally, there was no correlation between the number of total OLP responses (against all 410 OLP) and the magnitude of responses to beneficial OLP in either cohort, indicating that the strength of beneficial OLP responses was not diminished by other responses to the rest of the viral proteome.

**Table 2:** Beneficial and non-beneficial OLP identified in Durban clade C cohort (q < 0.2)

OLP #	Protein	Sub-unit	OLP clade C consensus sequence	Median viral load in OLP responders	Median viral load in OLP non-responders	Protective Ratio (PR)*	p-value	Q-value
3	Gag	p17	EKIRLRPGGKKHYMLKHL	18,700	45,100	**1.09**	0.0002	0.0006
6	Gag	p17	ASRELERFALNPGLL	6,570	44,100	**1.22**	0.0000	0.0000
7	Gag	p17	ERFALNPGLLETSEGCK	5,270	43,900	**1.25**	0.0000	0.0000
22	Gag	p24	WVKVIEEKAFSPEVIPMF	8,360	42,850	**1.18**	0.0000	0.0000
25	Gag	p24	GATPQDLNTMLNTVGGH	24,450	45,200	**1.06**	0.0021	0.0263
26	Gag	p24	NTMLNTVGGHQAAMQMLK	5,310	39,600	**1.23**	0.0061	0.0766
27	Gag	p24	GGHQAAMQMLKDTINEEA	9,715	42,100	**1.16**	0.0015	0.0170
29	Gag	p24	AAEWDRLHPVHAGPIA	19,700	40,900	**1.07**	0.0045	0.0544
31	Gag	p24	IAPGQMREPRGSDIA	6,480	38,950	**1.20**	0.0146	0.1478
33	Gag	p24	SDIAGTTSTLQEQIAWM	11,650	40,900	**1.13**	0.0025	0.0318
37	Gag	p24	WIILGLNKIVRMYSPVSI	9,360	44,100	**1.17**	0.0004	0.0018
39	Gag	p24	SILDIKQGPKEPFRDYV	2,630	38,250	**1.34**	0.0182	0.1838
41	Gag	p24	YVDRFFKTLRAEQATQDV	22,150	44,100	**1.07**	0.0020	0.0263
42	Gag	p24	LRAEQATQDVKNWMTDTL	16,480	40,900	**1.09**	0.0078	0.0935
55	Gag	p15	HIARNCRAPRKKGCWK	7,550	39,700	**1.19**	0.0092	0.1047
59	Gag	p15	RQANFLGKIWPSHKGR	9,840	42,200	**1.16**	0.0046	0.0539
60	Gag	p15	GKIWPSHKGRPGNFLQSR	6,130	39,700	**1.21**	0.0066	0.0799
63	Gag	p15	TAPPAESFRFEETTPAPK	6,040	38,950	**1.21**	0.0093	0.1020
116	Tat	Tat	TKGLGISYGRKKRRQRRS	109,000	36,700	0.91	0.0033	0.0410
178	Pol	RT	FWEVQLGIPHPAGLKKKK	258,000	37,300	0.84	0.0033	0.0384
181	Pol	RT	LDVGDAYFSVPLDEDFRK	7,100	38,950	**1.19**	0.0186	0.1832
190	Pol	RT	RAQNPEIVIYQYMDDLYV	84,900	34,700	0.92	0.0043	0.0555
199	Pol	RT	TVQPIQLPEKDSWTVNDI	6,700	38,300	**1.20**	0.0198	0.1926
216	Pol	RT	QKIAMESIVIWGKTPKFR	18,150	43,000	**1.09**	0.0026	0.0317
239	Pol	RT	QVDKLVSSGIRKVLFL	373,200	37,700	0.82	0.0205	0.1937
253	Pol	Int	PAETGQETAYFILKLAGR	92,800	35,400	0.92	0.0082	0.0954
265	Pol	Int	AVFIHNFKRKGGIGGYSA	63,650	33,800	0.94	0.0178	0.1826
283	Vpr	-	GLGQYIYETYGDTWTGV	78,000	35,600	0.93	0.0126	0.1302
284	Vpr	-	ETYGDTWTGVEALIRIL	85,050	35,200	0.92	0.0099	0.1034
312	Env	Gp120	YALFYRLDIVPLNENNSSEY	270,000	37,700	0.84	0.0208	0.1915
365	Env	Gp41	GIKQLQTRVLAIERYLK	151,000	34,700	0.88	0.0001	0.0002
393	Env	Gp41	LLGRSSLRGLQRGWEALKYL	750,000	37,450	0.78	0.0007	0.0041
417	Vif	-	CFADSAIRKAILGHIV	1,110	38,200	**1.50**	0.0178	0.1891

* PR values in bold indicate PR > 1, i.e. OLP-responses seen more frequently in individuals with reduced viral loads

In the clade B cohort, the 26 beneficial and 17 non-beneficial OLP showed a significant difference in their median entropy (p = 0.0327, Figure 2C), in line with the overall negative association between higher PR and lower sequence entropy seen in the comprehensive screening including the entire 410 OLP set (Figure 1C). While this comparison was not significant in clade C infection, a detailed look at Gag showed that beneficial Gag clade C OLP had a lower entropy values than the rest of the Gag OLP, suggesting that targeting of the most conserved regions even in Gag provided particular benefits for viral control (Figure 2D, p = 0.0172). These beneficial OLP were also more frequently targeted (median of 36 responders) compared to the rest of Gag OLP (median 12 responders, p = 0.0099), likely reflecting the high epitope density in these regions [33,44].

Finally, the two cohorts showed a partial overlap in the targeted beneficial and non-beneficial OLP, despite the vastly different HLA genetics in these two populations [4,31,45,46]. As Gag was enriched in beneficial OLP scattered throughout the entire protein sequence, we used the available reverse transcriptase (RT) protein structure to assess whether beneficial responses were targeting structurally related regions of the protein, even though the linear position of beneficial OLP did not precisely match between the two clades. Indeed, superimposing the locations of beneficial OLP in the RT protein indicates that in both clades, beneficial OLP fell in structurally related domains of the RT protein (Figure 2E and 2F). This suggests that despite differences in response patterns between ethnicities and clades, viruses from both clades may be vulnerable to responses targeting the same structural regions of at least some of their viral proteins.

### Increased breadth of responses against beneficial OLP is associated with decreasing viral loads, independent of Gag-specificity or the presence of protective HLA class I alleles

**Figure 3:**
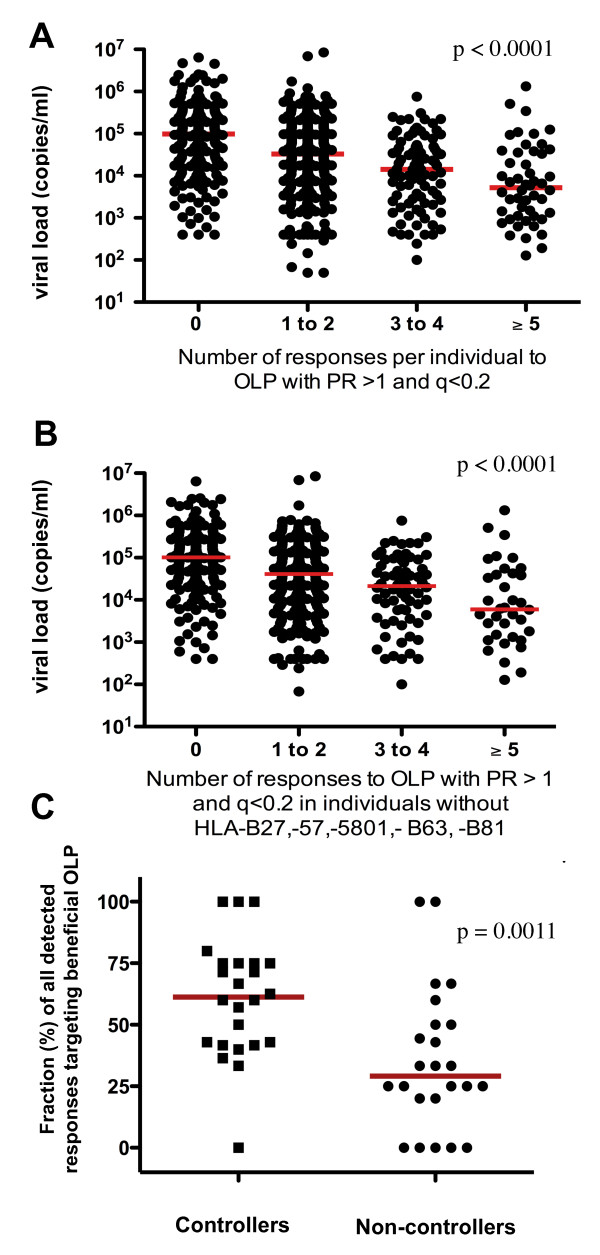
**Increased breadth of responses to beneficial OLP results in gradually reduced viral loads and is independent of cohort and HLA-B27, -57, -B58, -B81 and -B63**. (**A**) The number of responses to beneficial OLP in the clade C cohort in Durban was determined for each individual and compared to viral loads. An increased breadth of responses to the 22 beneficial OLP was associated with reduced viral loads (ANOVA, p < 0.0001). (**B**) This association remained equally stable after removing all individuals expressing known beneficial HLA allele (HLA-B27, -B57, -B5801, -B63, -B81) from the analysis (ANOVA, p < 0.0001). (**C**) The set of 26 beneficial and 17 non-beneficial OLP identified in the clade B infected cohort in Lima, Peru was tested in a second clade B infected cohort in Barcelona. HIV controllers showed a significantly higher focus of responses on the 22 beneficial OLP (61% of all responses to the 43 OLP) while non-controllers reacted predominantly with the non-beneficial OLP (only 29% of all responses targeting beneficial OLP). The Barcelona cohort did not included subject expressing any HLA allele previously associated with relative control of HIV-1 (p = 0.0011, Mann Whitney).

To assess whether individuals targeting more than one beneficial OLP profit from a greater breadth of responses to these targets, subjects in both cohorts were stratified by the number of responses to beneficial OLP and their viral loads compared. In both cohorts, negative correlations between the number of responses to beneficial OLP and viral loads were observed (p < 0.0001, r = -0.33 for Lima; p < 0.0001, r = 0.-25 for Durban; data not shown), suggesting that there is a cumulative benefit of responses to these particularly effective targets. Similarly, when individuals in the clade C cohort were grouped based on mounting 1-2, 3-4 or five and more beneficial OLP responses, a gradual reduction in median viral loads was seen. This reduction was close to 20-fold when 5 or more of the 22 beneficial OLP were targeted (median viral load 5,210 copies/ml) compared to individuals without a response (98,800 copies/ml, Figure 3A). Importantly, this observation was not driven only by individuals expressing HLA class I alleles associated with relative control of viral replication (including HLA-B27, -B57, -B*5801, -B63 and -B81) as their exclusion still showed a strong association between increased breadth of responses to beneficial OLP and a gradual suppression of viremia (Figure 3B). This was further supported when translating the clade B data from Peru to a second clade B infected cohort in Barcelona, Spain where HIV-1 controllers also mounted a significantly greater proportion of their responses to the beneficial Peruvian OLP compared to the HIV-1 non-controllers (61% vs. 29%, p = 0.0011; Figure 3C); this despite the fact that the Barcelona cohort was genetically different and excluded individuals expressing HLA-B27, -B57, -B58 and B63. Thus, despite the frequent targeting of Gag and the inclusion of individuals expressing HLA alleles such as HLA-B*5701 and -B*5801 in the two larger clade B and C cohorts, the present data identify regions of the viral genome that serve as the targets of an effective host T cell response, largely independent of the presence of HLA alleles known to influence HIV-1 viral replication.

### PR-values are mediated by individuals with broad HLA heterogeneity

To further assess the contribution of specific HLA class I alleles on the PR of individual OLP, the statistically significant OLP in the clade C cohort were further analyzed. In a first step, median viral loads in the OLP-responder and non-responder groups were compared after excluding individuals with specific HLA class I alleles. If the statistical significance of the comparison was lost, the excluded HLA class I allele was assumed to have significantly contributed to the initially observed elevated or reduced PR value and to restrict a potential CTL epitope in that OLP. In a second step, a "Hepitope" analyses http://www.hiv.lanl.gov/content/immunology/hepitopes was conducted to identify HLA class I alleles overrepresented in the OLP responder group; providing an alternative approach to identify specific epitopes that may contribute to relative viral control. Together, the two strategies permit to estimate the HLA diversity in the OLP responders and to identify the most likely alleles that restrict the epitope-specific responses to the OLP. Both are important measures when determining the relative usefulness of a selected beneficial OLP in a potential immunogen sequence as it should provide broad HLA coverage. The data from these analyses are summarized for beneficial and non-beneficial OLP in Table 3 and 3, respectively. The results demonstrate that with a few exceptions, for each OLP, several HLA alleles appeared to be mediating the observed effects as their removal caused the statistical significance to be lost. However, for the most frequent HLA class I alleles, the loss of significance may be due to a reduction in sample size rather than the actual allele, since the exclusion of many allele carriers could reduce the number of OLP responders (and non-responders) sufficiently to lose statistical power. The "Hepitope" analysis controlled for this effect and confirmed the obtained results, strongly indicating that responses to beneficial OLP were mediated by responder populations with heterogeneous HLA allele distributions.

**Table 3:** Impact of HLA alleles on the statistical significance of observed PR values (clade C OLP)

A) Beneficial OLP (PR > 1)				
OLP	Protein	PR	**Removed HLA allele(s) abolishing statistical significance**^**1**^	**Alleles over-represented in the OLP responder group**^**2**^
3	Gag	1.09	A30, B42, C17	A30, B08, A03, A74, C17, A43, B42, B07
6	Gag	1.22	*B15*	B49, B82, C14
7	Gag	1.25	-	B42, C17, B49, A30
22	Gag	1.18	B57, C07	B57, A74, B45, C07, C16, B13
25	Gag	1.06	A30, *B15, C04, C07*	B42, C17, B81, B39, A01, C12, C18, A30, B67
26	Gag	1.23	*A02, A23*, A68, *B07, B14, B58, C07, C08*	C03, B15, A68
27	Gag	1.16	B15, *C07*	B15, A68, C03, C08
29	Gag	1.07	*A68, B15, B58, C02, C03, C06*, C12	B35, B39, C12, B40, B07, C04
31	Gag	1.2	*A02*, A11, *A23*, A29, *A32, A34, A68, B07*,	B13, A29, C06, A11
			B13, B15, B42, B44, B58, C04, C06, C07, C17	
33	Gag	1.13	A02, *A23, B44*, B57, B58, C07	B58, B57, A02, C07, C03, A68
37	Gag	1.17	*A30*, B42, *B58*, C17	C18, B42, C17, A01, B81
39	Gag	1.34	A02, *A03, A23, A29, A30, A68, A74, B08, B15*,	A02
			B18, B42, B45, B53, B57, B58, C02, C03, C06,	
			C07, C08, C16, C17	
41	Gag	1.07	*A23, C06*	C03, B14, A68, C08, B15
42	Gag	1.09	*A23, A30, B08, B15, B42*, B53, *B58*, C03, *C04, C07*	B53, C03
55	Gag	1.19	*A02, A24, A29, A30, B07, B15, B39*, B42, *B44*,	B42, B08, C17
			B58, C02, C06, C07, C17	
59	Gag	1.16	A02, *A30, B08, B42, B44, B58, C04, C07, C17*	A02, B13, A29
60	Gag	1.21	A02, *A30, B42, B58, C06*, C07, C17	A02, B41, C07, C17
63	Gag	1.21	*A02*, A23, *A29, A30, A68, B08, B15, B44, B58*,	A23
			C02, C03, C06, C07	
181	Pol	1.19	*A01, A23, A29, A30, A34, A68, A74, B14, B15*,	B57, C18
			B18, B35, B44, B45, B57, B58, C02, C03, C04,	
			C06, C07, C08, C16	
199	Pol	1.2	*A02, A03*, A23, *A24, A26, A30, A31, A34, A36, A66, A68*,	B53, A23, C04
			A80, B08, B13, B15, B18, B35, B40, B41, B42, B44, B45,	
			B49, B50, B51, B53, B57, B58, B81, C01, C02, C03, C04,	
			*C05, C06, C07, C08, C15, C16, C17*	
216	Pol	1.09	*A02, A30*, B58, C07, *C17*	B53, B58, C07, B57
417	Vif	1.5	*A03, A23, A30, A34*, A36, *A68, B08*, B14, *B15*,	B14, C08, A36
			B44, B53, B58, C03, C04, C06, C08	
**B) Non-beneficial OLP (PR < 1)**				
116	Tat	0.91	*A02, A34*, B15, *C04*	B15, C02
178	Pol	0.84	A03, A68, *B15*, B58, *C04*, C06, *C07*	A68, C06, B58, B82, A03
190	Pol	0.92	*A03, A30, A66*, B18, *B42*, B45, *B58, C06, C07*	A02, B18, B35, C05, C16, B45, A80, C12, B67, B39
239	Pol	0.82	-	C05, A03
253	Pol	0.92	*A03*, A68, B15, *B39, B42, B44, B58, C02, C04, C06, C08*,	A68, C03, B15, B07, C15, B41
			C17, C18	
265	Pol	0.94	*A02, A03, A23, A24, A26, A29, A30, A31, A33*,	B15, C02, A43, A74
			*A34, A66, A68, B07, B08, B14, B15, B27, B40, B41, B42*,	
			*B44, B53, B54, B55, B57, B81, C01*, C02, *C04, C06, C07*,	
			*C12, C17, C18*	
283	Vpr	0.93	*A02, A03, A23, A30, A66*, A68, *A74*, B07, *B14, B18, B39*,	A68, C03, B07, C17, B41
			*B41, B42, B45, B57, C02, C04, C07, C08, C15, C17*	
284	Vpr	0.92	*A03, A23, A30, A66*, A68, *A74, B07, B14, B18, B39, B42*,	A68, C03
			*B45, B57, C07, C08, C15, C17*	
312	Env	0.84	-	B08, C07
365	Env	0.88	B58, C06	C06, B58, A43, B45, C16, A66
393	Env	0.78	*A30, B58*, C06	A31, C06, B45

1) in italics HLA alleles that do not emerge from the Hepitope analysis 2) cut-off in Hepitope analyses for p < 0.05, alleles sorted according to strength of association

### Effects of T cell specificity on in vivo viral load are at least as strong as those associated with host HLA genetics

**Figure 4:**
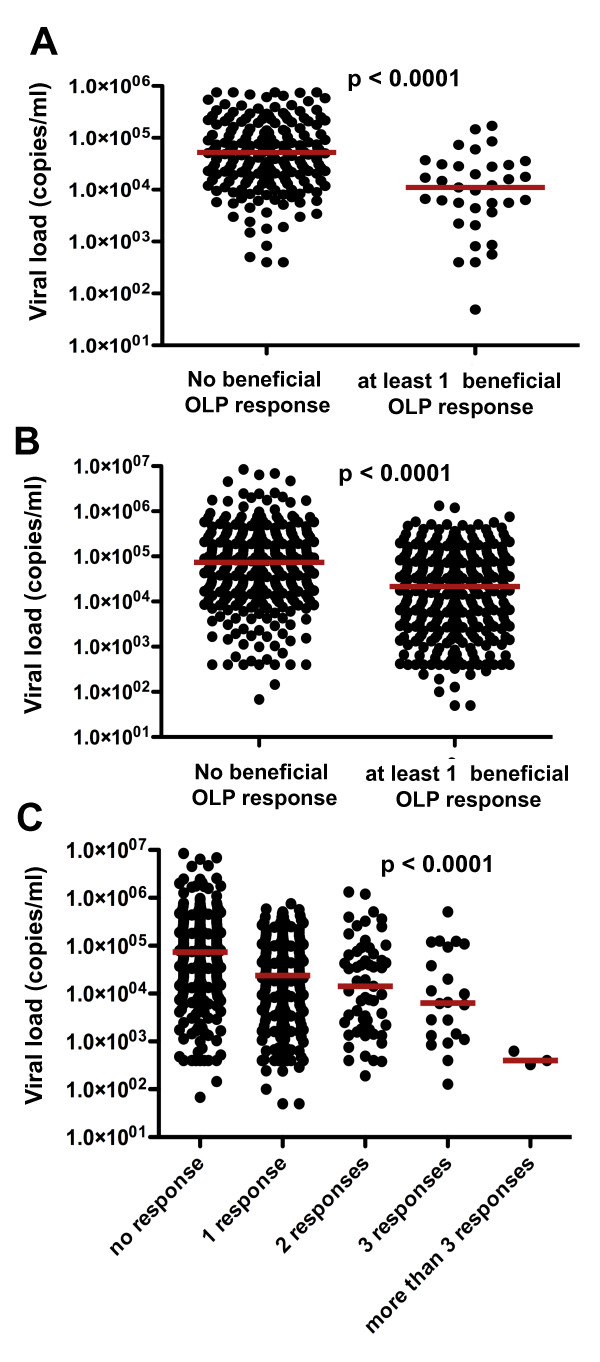
**Responses to OLP identified in multi-variate analysis are associated with reduced viral loads**: Response patterns and HLA class I genetics in the clade B cohort in Lima and clade C cohort in Durban were subjected to FASS multivariat analysis [41-43]. Viral loads in individuals mounting zero vs. at least one response to beneficial OLP identified by the FASS multi-variate analysis were compared for (**A**) the Lima clade B cohort and the (**B**) Durban clade C cohort. The larger data set for the clade C cohort allowed for a further stratification of the responder group by increasing numbers of targeted OLP emerging from the FASS analysis (**C**). A gradually declining median viral load in relation to an increasing breadth of these responses was seen (ANOVA, p < 0.0001).

To assess whether specific response patterns and/or HLA combinations could be identified that mediated synergistic or superior control of viral infection in clades B and C, multivariate combined regression analysis was conducted on either OLP only, HLA only or the combination of OLP and HLA variables [41-43]. The OLP-only analysis for Lima identified 7 OLP of which 4 were associated with lower median viral loads and 3 with increases in viral loads, respectively (Table 4). Targeting at least one of these beneficial clade B OLP was associated with significantly reduced viral loads (median 11, 079 copies/ml) compared to the subjects who did not target any of these four OLP (median 52, 178 copies/ml; p < 0.0001, Figure 4A). As seen in the univariate analysis (Figure 2C), the four beneficial OLP emerging from the Lima FASS analysis were more conserved than the rest of the OLP (median entropy 0.0759 vs. 0.1649, p = 0.0267) or the three non-beneficial OLP (0.0759 vs. 0.1228, p = 0.0571, data not shown). In contrast to OLP-only FASS analysis, only one HLA allele (HLA-C04) emerged from the HLA-only multivariate analysis. The analysis for the combined variables (OLP and HLA) controlled for the potential bias in this result due to more OLP variables (n = 389) than HLA (n = 146) being included in the statistical tests; yet still identified more OLP variables (n = 9) than HLA class I alleles (n = 3). In addition, the relative co-efficients of these associations were stronger for the OLP than the HLA variables, suggesting that T cell specificity influenced viral loads to at least the same degree as host HLA class I genetics. Of note, the identified OLP and HLA variables did not reflect responses to known optimal CTL epitopes, as none of the OLP contained described epitope(s) restricted by any of the identified HLA alleles [44].

**Table 4:** Multivariate analysis of OLP and HLA variables for clade B and C cohorts

OLP variables only (Lima, clade B)			OLP variables only (Durban, clade C)		
	change viral load (co-efficient) *	p-value		change viral load (co-efficient) *	p-value
Beneficial			Beneficial		
OLP.6	-0.4591	0.0008	OLP.7	-0.6256	0
OLP.31	-1.4055	0.0002	OLP.21	-0.6663	0
OLP.171	-2.5981	0	OLP.22	-0.4926	0.0006
OLP.276	-1.127	0.0007	OLP.25	-0.2822	0.0002
			OLP.27	-0.4719	0.0053
Non-beneficial			OLP.33	-0.3396	0.0024
OLP.76	0.2486	0.0067	OLP.398	-1.8179	0.0027
OLP.306	3.2968	0.0001	OLP.417	-1.6535	0.0008
OLP.411	1.3329	0.012			
			Non-beneficial		
			OLP.38	0.9045	0.0022
			OLP.84	0.1947	0.0091
			OLP.116	0.6156	0.0039
			OLP.183	0.661	0.0013
			OLP.224	0.2508	0.0036
			OLP.265	0.5782	0
			OLP.365	0.4911	0.0009
			OLP.393	1.2624	0.0013
**HLA variables only (Lima, clade B)**			**HLA variables only (Durban, clade C)**		
Non-beneficial			Beneficial		
HLA-C0401	0.35652	0.00024	HLA-A74	-0.3553	0.0025
			HLA-B13	-0.6443	0.0004
			HLA-B57	-0.5195	0.0007
			HLA-B81	-0.3619	0.0015
			HLA-C12	-0.6544	0.0001
			Non-beneficial		
			HLA.B.15	0.2506	0.0012
			HLA.B.18	0.5521	0.0005
			HLA.C.6	0.3958	0
					
**HLA and OLP variables together (Lima)**			**HLA and OLP variables together (Durban)**		
Beneficial			Beneficial		
OLP.6	-0.5792	0	OLP.6	-0.4798	0.0023
OLP.31	-1.1607	0.0005	OLP.7	-0.4528	0.0015
OLP.171	-2.7948	0	OLP.27	-0.4676	0.0049
OLP.276	-0.9609	0.0011	OLP.59	-0.4196	0.0115
			OLP.417	-1.387	0.0041
Non-beneficial			Non-beneficial		
OLP.2	0.3945	0.018	OLP.148	2.5215	0.0029
OLP.237	0.7211	0.0035	OLP.183	0.6108	0.0023
OLP.288	1.5537	0.0016	OLP.393	1.1442	0.0023
OLP.311	0.7197	0.0091			
OLP.411	1.5306	0.0024	Beneficial		
			HLA-A74	-0.3744	0.0007
Beneficial			HLA-B57	-0.4887	0.0007
HLA-B1502	-1.4688	0.0164	HLA-B81	-0.3859	0.0004
			HLA-C12	-0.6003	0.0002
Non-beneficial			Non-beneficial		
					
HLA-B0801	0.66	0.0049	HLA-B15	0.2797	0.0001
HLA-C0401	0.2894	0.0006	HLA-B18	0.5316	0.0003
			HLA-B49	0.9713	0.0007

* negative co-efficient values indicate reduction in median viral loads

Results from the clade C cohort in Durban confirmed the clade B findings in Lima as the FASS analyses identified 16 OLP but only 8 HLA variables that had an impact on the individual viral loads. As in Lima, the impact of OLP specificity was at least as strong than HLA genotype (trend for higher coefficients for OLP than HLA; data not shown, p > 0.05). In addition, targeting at least one of the eight beneficial OLP in Durban was associated with strongly reduced viral loads (p < 0.0001, Figure 4B). This effect was, as in the univariate analysis, additive for more than one response (p < 0.0001, Figure 4C) and included OLP that were, aside from Gag, located in Pol and Vif. Also, the combined (OLP and HLA) analysis suggests the effect of OLP specificity on viral loads to be at least as strong as HLA genetics as 8 OLP and 7 HLA variables were identified. This especially since among the 7 HLA alleles, two (HLA-B57 and HLA-A74) are expressed in linkage disequilibrium [47], further reducing the number of HLA variables with a significant impact on viral loads.

### Responses to beneficial OLP are of higher functional avidity and suppress viral replication in vitro more effective than responses to non-beneficial OLP

**Figure 5:**
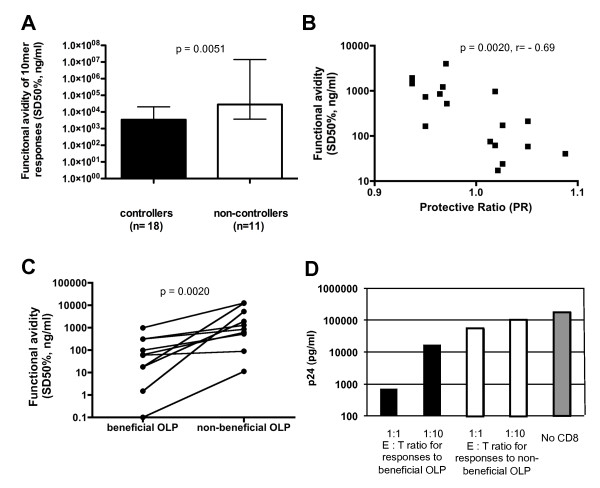
**Responses to beneficial OLP are of higher functional avidity and suppress in vitro viral replication more effectively**. (**A**) Responses to the four beneficial OLP located in HIV-1 clade B Gag p24 were retested using a peptide set of 10 mers overlapping by 9 residues. A total of 21 responses in HIV-1 controllers and 24 responses in HIV-1 non-controllers were titrated and the SD50% compared between the two groups, showing a significantly higher functional avidity in the controllers (p = 0.0051, Mann Whitney). (**B**) Responses to 17 different optimally defined CTL epitopes located in beneficial, neutral and non-beneficial OLP were titrated in samples from 78 HIV infected individuals with variable viral load and disease status. The median SD50% (ng/ml) was defined for each epitope and compared to the OLP-specific protective ratio (Spearman Rank test, p = 0.0020). (**C**) Ten individuals who mounted responses to well-defined optimal CTL epitopes located in beneficial as well as in non-beneficial clade B OLP were identified and their responses titrated. The SD50% for responses detected in the same individual were compared (Wilcoxon matched pairs test, p = 0.0039). (**D**) In-vitro viral replication inhibition assays [48] were performed using a Nef modified and Raltegravir resistant test virus and purified CTL effector populations from the same individual targeting beneficial and non-beneficial OLP. One representative experiment of three assays conducted in different individuals is show. Levels of Gag p24 were determined after 4 days of co-culture of effector cells and auologous CD4 T cells used as target cells. Target cells were stimulated 3 days prior with dual-specific anti-CD3/8 mAb and infected at a MOI of 0.1. The negative control contained wells with target cells only ("no CD8").

Functional avidity and the ability to suppress in vitro viral replication have emerged as two potentially crucial parameters of an effective CTL response against HIV-1 [23-29]. To assess this potential functional characteristic of beneficial CTL populations, we determined the functional avidity of responses to the four beneficial OLP located in Gag p24, a region that has been most consistently associated with eliciting relatively protective CTL responses. As 18 mer peptides are suboptimal test peptides to determine functional avidity, 10 mer overlapping peptide sets were synthesized to cover the four beneficial OLP and all detected responses were titrated. The SD50% was determined for a comparable numbers of responses detected in controllers (n = 21 responses) and non-controllers (n = 24 responses) and showed a statistically significant difference between the two groups (median 3, 448 ng/ml vs. 25, 924 ng/ml, p = 0.0051, Figure 5A). This reduced avidity in HIV non-controllers to beneficial OLP could possibly explain why HIV-1 non-controllers did not control their in vivo viral replication despite targeting these regions in some instances and with responses of comparable magnitude as HIV controllers (278 SFC vs 305 SFC/10^6 ^PBMC, p = 0.55, data not shown).

To more directly assess whether responses to beneficial OLP were of particularly high functional avidity, regardless of HIV controller status, we determined SD50% of responses to 17 optimal epitopes from beneficial, neutral and non-beneficial OLP (Figure 5B). Median epitope-specific SD50% were determined from an average of 7 titrations per epitope and compared to the OLP specific PR. A strongly significant, negative association between the PR and the SD50% was noted (p = 0.002, r = -0.69), indicating that beneficial OLP are targeted by high-avidity responses. To control for inter-individual differences due to disease status and viral load, we identified 10 individuals who targeted optimal epitopes in beneficial and non-beneficial OLP and determined their functional avidity. As in the cross-sectional analysis before, this matched comparisons showed in all cases a higher functional avidity for the epitopes located in the beneficial OLP compared to the responses targeting non-beneficial OLP (Figure 5C, p = 0.0020). Lastly, to relate the higher functional avidity to potential superior anti-viral effects in vivo, the ability to inhibit in vitro viral replication was assessed in three individuals who mounted robust responses against both beneficial and non-beneficial OLP. The in vitro inhibition assay first developed by Yang et al [48], was modified so that the NL4-3 based test virus contained a single nucleotide mutation in Nef (M20A) that blocks the Nef-mediated down-regulation of HLA class I molecules as well as two mutations in the integrase gene that mediate Raltegravir-resistance to permit the suppression of potentially replicating autologous virus in the assay. Indeed, CTL specific for the beneficial OLP(s) were up to 2 logs more effective inhibiting viral replication than CTL targeting non-beneficial OLP (Figure 5D), in line with recent data demonstrating different suppressive ability of HIV-1 specific CTL populations targeting Gag and Env-derived epitopes [24]. Although the in vitro inhibition assays were limited to few individuals with suitable response patterns, these data together with the results from the extensive titration assays in Figure 5B and 5C indicate that responses to beneficial OLP are of particularly high functional avidity and inhibit in vitro viral replication more effectively than responses to non-beneficial OLP. Of note, higher avidity responses to beneficial OLP compared to non-beneficial OLP were seen in all 10 tested individuals, ruling out that inter-individual variability in viral loads, duration of infection and HIV disease status could have biased the analyses.

## Conclusions

Defining functional correlates of HIV-1 immune control is critical to the design of effective immunogens. T cell responses to specific HIV-1 proteins and protein-subunits have been associated before with relatively superior viral control in vivo [14,16,49], but evidence from recent clinical trials suggests that including maximal immunogen content into various vectors does not necessarily induce more effective CTL responses [50,51]. In fact, it has been argued that the existence of potential "decoy" epitopes may divert an effective CTL response towards variable and possibly less effective targets in the viral genome [52]. Thus, the definition of a minimal yet sufficient immunogen sequence that can elicit CTL responses in a broad HLA context is urgently needed. Thereby, focusing vaccine responses on conserved regions could help induce responses towards mutationally constrained targets and provide the basis for protection from heterologous viral challenge.

We present here the results of an extensive analysis that included more than 950 HIV-1 infected individuals with diverse HLA genotypes, from three different continents and including clade B and C infections. In both, the analysis in clade B in Lima and clade C in Durban, individual OLP were identified that are predominantly targeted by individuals with reduced or elevated viral loads, although the different size of the cohorts required different statistical approaches for their identification. In general, most of these OLP were among the more frequent targets in the HIV proteome, possibly due to both, the need for sizable responder groups to achieve statistical significance in the viral loads comparison as well as the high epitope density in these OLP. The identified OLP were frequently located in HIV-1 Gag and Pol, but rarely in the more variable proteins such as Env and Nef. With one exception, Nef and Env featured only non-beneficial OLP, thus arguing against their inclusion, at least as full proteins, in a CTL immunogen sequence [16]. In addition, in both cohorts, the Vif protein yielded few, yet exclusively beneficial OLP, which may warrant a renewed look at the inclusion of regulatory proteins in vaccine design [53,54]. Also common to both clades, (and despite the wide scatter possibly due to the inclusion of less-frequently targeted OLP), an negative correlation between sequence entropy and PR was observed providing strong rationale for vaccine approaches that focus on conserved viral regions where T cell escape may be complicated by structural constrains [55]. This was particularly evident in the clade C cohort, where even within the relatively conserved Gag protein, a lower entropy was seen for the beneficial OLP compared to the remainder of the OLP spanning the protein. On the other hand, while beneficial and non-beneficial OLP showed a significant difference in their median entropy in the clade B cohort, this comparison was not significant in the clade C cohort. It is possible that the immunogen sequence, designed in 2001, did not optimally cover the circulating viral population in Durban throughout the enrollment period (until 2006), leading to missed responses particularly in the more variable segments of the virus [32,56]. The study may have thus failed to identify beneficial as well as non-beneficial OLP in the more variable genes of HIV. This should have preferentially affected highly variable OLP due to a more frequent mismatch between autologous viral sequence and in vitro test set in these regions. However, even if scoring as beneficial OLP, such high-entropy OLP may from an immunogen-design point of view be of less interest as they would possible contribute only little to protection from heterologous viral challenge. It needs however also to be considered that the OLP-specific entropy values are based on variable numbers of sequences in the Los Alamos HIV database covering the different OLP, introducing potential further bias into these analyses, particularly for less covered proteins such as Vpu and other viral protein products. Such differences between autologous viral sequences and in vitro test sets may also have impacted the assessment of functional avidities. These determinations included responses in the same individual towards epitopes located in beneficial and non-beneficial OLP; with the former overall being more conserved. Thus, the higher functional avidity towards epitopes located in beneficial OLP could be biased by the higher chance that these epitopes matched the autologous viral sequence compared to epitopes located in non-beneficial OLP and which may thus have induced a more robust, avid response. Apart from covering autologous sequences, future studies will ideally also include comparable analyses in individuals identified and tested in acute infection that go on to control the infection at undetectable levels of viral replication (i.e. elite-controllers) so that the selective early emergence of responses to beneficial OLP could be linked to relative control of viral replication in chronic infection. As is, the identified beneficial responses may be particularly important to maintain low viral replication in chronic stages of infection, which in theory could be different (for instance due to more accelerated intra-individual viral evolution in variable genes) from responses determining viral set point during acute infection. However, the existing HLA bias in such cohorts and the small number of responses identified during earliest stages of infection may make such analyses a formidable undertaking that will require large numbers of individuals to be tested longitudinally.

A broadly applicable T cell immunogen sequence should include T cell targets restricted by a wide array of HLA class I alleles. Although broad representation of HLA-B alleles may be particularly important in this regard, emerging data on the effects HLA-C alleles in these cohorts may warrant a broad HLA-C representation as well [2,47,57]. In the present study, the 26 beneficial OLP from Lima and the 22 beneficial OLP from Durban covered 26 described, optimally defined CTL epitopes restricted by 20 different HLA alleles for the clade B cohort and 33 epitopes presented by 34 alleles for the clade C cohort, respectively [44]. As this is likely to be an underestimate of the true diversity in HLA restriction (Table 2 and ref [58]), it is reasonable to predict that the inclusion of identified beneficial OLP, or even a subset thereof, could evoke potential responses in a widely diverse HLA context. This could also provide the basis for the induction of poly-specific T cell responses with increased breath, which the present data clearly associates with progressively lower viral loads and which emerge as a potentially important parameter from several recent vaccine studies showing superior protection from SIV challenge in animals with a broad vaccine induced responses to Gag p17 [59,60].

Recent studies have suggested a global adaptation of HIV-1 to its various host ethnicities [4,46]. The consequence of such adaptation has led in some cases to the elimination of protective CTL targets, causing a profound absence of responses to these epitopes and detrimentally changing the association between HLA allele and HIV-1 disease outcome [4]. It is thus not surprising that the two main cohorts tested here yielded only partially overlapping sets of beneficial OLP as the impact of host genetics and viral evolution in the studied populations cannot readily be overcome. In fact, given studies by Frahm et al [4], the past and current adaptation of HIV-1 to common HLA class I alleles will likely still call for somewhat population tailored vaccine approaches, especially if the immunogen sequences should be kept short to avoid regions of potentially reduced immunological value [52]. Such approaches will also profit from more extensive structural analyses that may identify specific domains of viral proteins that are or are not enriched in valuable T cell targets; of which the latter could possibly be ignored for the design of T cell immunogen sequences. Additional analyses in other genetically unrelated cohorts of HIV-1 infected individuals and studies in SIV infection may further help to guide such selective immunogen design and to understand the factors defining the effectiveness of different epitopes in mediating relative HIV-1 control. Of note, the beneficial OLP identified here, 24 in clade B and 22 in clade C infection matched other immunogen design based on conserved elements in some parts as well, i.e. of the 14 conserved elements proposed by Hanke et al, eight (57%) overlapped at least partly with beneficial OLP identified here [61]. Similarly, among the highly conserved elements proposed by Rolland et al [52], 35% (5/14) were covered by our beneficial OLP in clade B infection. These differences possibly emerge because the present analysis is based on functional T cell data rather than viral sequence alignments, which may not take into consideration epitope density and processing preferences of certain regions. Nevertheless, the partial overlap with these other immunogen design support the focus on conserved regions and offers the opportunity for alternative or combined vaccine approach that elicit responses to regions where the virus is and possibly remains vulnerable [4,46,55,62].

Finally, we used the extensive data set available to approach the question of relative effects of host genetics (i.e. HLA) and CTL specificity on HIV-1 control. While the two factors cannot be entirely disentangled, our data suggest that CTL specificity has an at least equal if not stronger effect on viral control than HLA class I allele expression. These findings are also in line with data by Mothe et al [63] showing that targeting key regions in p24 surrounding the dominant epitopes restricted by known protective alleles (KK10 for HLA-B27 and TW10 for HLA-B57/58) in HLA-B27, -57 or B58 negative individuals is associated with significantly reduced viral loads. In addition, the presence of individuals not expressing known beneficial alleles in HIV-1 elite controller cohorts [64], further indicates that HIV-1 control is not necessarily bound to a few specific HLA class I alleles. A detailed study of the total HIV-1-specific CTL response of subjects not expressing these alleles yet effectively controlling HIV-1 can be expected to provide further and crucially needed insight into the importance of targeting specific (conserved) regions of the viral genome for HIV-1 control. Similarly, the characterization of functional attributes of these responses, including functional avidity and the ability to suppress *in vitro *viral replication will need to be further assessed in such individuals. Building on experimentally derived and potentially promising immunogen sequences as defined here may thus provide a suitable basis for further immunogen design and iterative clinical trials in the human setting.

## Competing interests

The authors declare that they have no competing interests.

## Authors' contributions

BM conducted cellular immune analyses, in vitro inhibition analyses and drafted the first version of the manuscript. AL, JZ, VB generated the recombinant test virus, performed viral inhibition analyses and did the OLP screening of the Barcelona patients. JI and MD conducted OLP data analyses and HLA-epitope predictions. CM recruited patients and provided samples, RZ conducted and coordinated the screening of the Lima cohort subjects, SPA conducted statistical analyses and developed the multivariat analysis approach, CTB performed functional avidity analyses, MCP, JMP, OOY provided semi-genome plasmids and helped in the construction of the mutant test virus for inhibition analyses. MR, CJB, ZLB analyzed beneficial OLP sequences for HLA footprints associated with reduced viral fitness, MF conducted the screening of the Lima cohort subjects, JJS developed HEPITOPE tool and conducted HLA linkage analyses, WH performed HLA typing, VSM provided samples, DH performed initial multivariat analyses, TMA coordinated HLA typing and sequence analyses for the Lima cohort, JIM analyzed beneficial OLP sequences for HLA footprints and levels of sequence conservation, GG helped with the statistical analyses and the development of the multivariat analysis, PJG and BDW coordinated all OLP screenings, HLA typing and data collection in the Durban cohort, JMG and BC coordinated sample access and HLA typing in Barcelona, BTK helped with data analysis and writing of the manuscript, JS coordinated patient enrollment, ethical approval for the Lima cohort. CB conceived the study, conducted initial data analyses, and helped writing the manuscript. All authors were involved in the writing of the final manuscript and have given final approval of the version to be published.
